# Anthropogenic and climatic impacts on historic sediment, carbon, and phosphorus accumulation rates using ^210^Pb_ex_ and ^137^Cs in a sub-watershed linked to Zarivar Lake, Iran

**DOI:** 10.1007/s10661-024-13048-5

**Published:** 2024-09-04

**Authors:** Maral Khodadadi, Max Gibbs, Andrew Swales, Arsenio Toloza, William H. Blake

**Affiliations:** 1 Department of Geology and Environmental Earth Science, Miami University, Oxford, OH USA; 2 Nuclear Agriculture Research School, Nuclear Science and Technology Research Institute (NSTRI), Karaj, Iran; 3 National Institute of Water and Atmospheric Research (NIWA), Hamilton, New Zealand; 4 Soil and Water Management & Crop Nutrition Section and Laboratory, Department of Nuclear Sciences and Applications, Joint FAO/IAEA Division, International Atomic Energy Agency, Vienna, Austria; 5 School of Geography, University of Plymouth, Drake Circus, Plymouth, PL4 8AA UK

**Keywords:** Sediment chronology, Particulate P, Geochemical indices, Land-use change, δ^13^C, Inland lakes

## Abstract

To estimate a watershed’s response to climate change, it is crucial to understand how human activities and climatic extremes have interacted over time. Over the last century, the Zarivar Lake watershed, Iran, has been subjected to various anthropogenic activates, including deforestation and inappropriate land-management practices alongside the implementation of conservation measures like check dams. To understand the effects of these changes on the magnitude of sediment, organic carbon (OC), and phosphorus supplies in a small sub-watershed connected to the lake over the last century, a lake sediment core was dated using ^210^Pb_ex_ and ^137^Cs as geochronometers. The average mass accumulation rate (MAR), organic carbon accumulation rates (OCAR), and particulate phosphorus accumulation rates (PPAR) of the sediment core were determined to be 6498 ± 2475, 205 ± 85, and 8.9 ± 3.3 g m^−2^ year^−1^, respectively. Between the late 1970s and early 1980s, accumulation rates were significantly higher than their averages at 7940 ± 3120, 220 ± 60, and 12.0 ± 2.8 g m^−2^ year^−1^ respectively. During this period, the watershed underwent extensive deforestation (12%) on steep slopes, coinciding with higher mean annual precipitations (more than double). Conversely, after 2009, when check dams were installed in the sub-watershed, the sediment load to the lake became negligible. The results of this research indicate that anthropogenic activities had a pronounced effect on MAR, OCAR, and PPAR, causing them to fluctuate from negligible amounts to values twice the averages over the last century, amplified by climatic factors. These results imply that implementing climate-smart watershed management strategies, such as constructing additional check dams and terraces, reinforcing restrictions on deforestation, and minimum tillage practices, can facilitate protection of lacustrine ecosystems under accelerating climate change conditions.

**Graphical Abstract:**

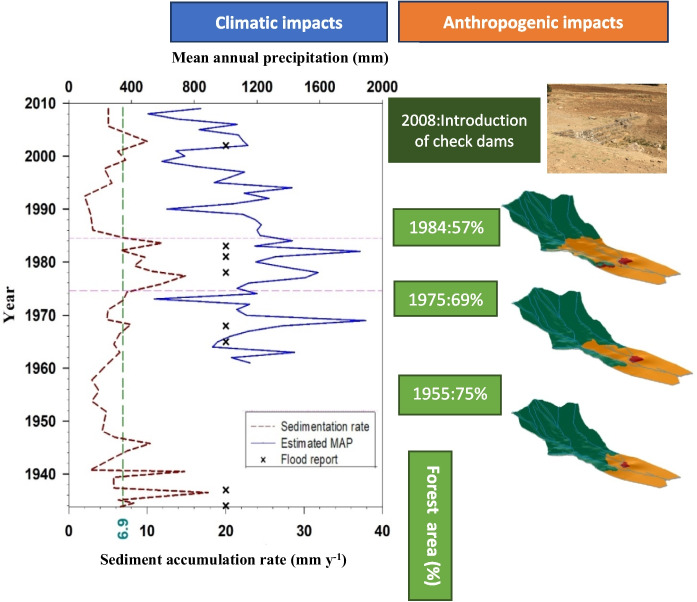

**Supplementary Information:**

The online version contains supplementary material available at 10.1007/s10661-024-13048-5.

## Introduction

The increased influx of fine sediments and their associated chemical constituents into reservoirs can indeed lead to adverse effects on water quality, aquatic life (Kemp et al., 2011), reservoir storage capacity (Kondolf et al., 2014), and primary production (Harmon et al., 2014). Climatic extremes (Kortelainen et al., 2013) and anthropogenic activities, particularly land-use change (LUC; e.g., Foster et al., 2003; Valero-Garcés et al., 2006; Anderson et al., 2013; Lin et al., 2021), are known to influence the amounts of fine sediments entering reservoirs. However, understanding the effect of human–environment interactions at the basin scale over historical times can be highly complex (Dearing et al., 2008). This complexity can be attributed to the limited availability of precise data on past climatic events and human activities, as well as the intricate interplay between human and climate influences. In this context, sediment cores can provide high-resolution temporal records of landscape response, aiding understanding how anthropogenic and climatic influences propagate through space and time (Canuel et al., 2017; Foster et al., 2003). Such knowledge can be applied to understand the current state of ecosystems (Zan et al., 2012) and predict the future ecosystem responses to climate change (Canuel et al., 2017), facilitating the implementation of effective conservation strategies and climate-smart agriculture practices to assure food security. In other words, reconstructing past environmental conditions enables informed future management decisions to protect and restore freshwater ecosystems in the face of increasing anthropogenic pressures and climate change.

Elevated dissolved organic carbon (OC) loadings to lakes cause heterotrophic conditions, where the rate of ecosystem respiration surpasses primary productivity, leading to a net efflux of CO_2_ into the atmosphere (Cole et al., 2007). In addition, lakes tend to accumulate large amounts of OC in the sediment, thus playing a vital role in the carbon cycle (Anderson et al., 2013; Kortelainen et al., 2004; Tranvik et al., 2009). The amount of accumulated OC in the sediment signifies carbon sequestration in short-to-long-term time scales (Downing et al., 2008). Organic carbon accumulation rate (OCAR) in lakes represents the difference between inputs from aquatic primary production and catchment fluxes and losses through respiration and outflow (Tranvik et al., 2009). Overall, understanding how OCAR may have changed over time, particularly in response to changes in land use and climate, could provide valuable insights into how future global change processes might affect OCAR in lakes (Anderson et al., 2013; Kortelainen et al., 2013; Lin et al., 2021) and, consequently, their storage capacity (Kondolf et al., 2014). Besides, excessively high concentrations of nutrients like nitrogen (N) and phosphorus (P) stimulate algal growth and are the most common cause of eutrophication in freshwater reservoirs (Sterner, 2008). P, in particular, is a limiting nutrient for primary production in freshwater ecosystems, playing a crucial role in influencing the trophic level of water bodies (Sterner, 2008). Additionally, in shallow eutrophic lakes, sediments, both internal sediment P and suspended particulate matter P, are the predominant sources of P (Radbourne et al., 2019), with particulate phosphorus (PP) constituting the largest portion of total phosphorus (TP; Yang et al., 2020). PP in lakes can originate from terrestrial sources or the decomposition of aquatic plants (Liu et al., 2020). The sediment-associated PP buried in deposited sediments can be released as soluble phosphate into the water column under anoxic conditions and contribute to eutrophication in water bodies (Heathcote et al., 2015). These factors underscore the importance of identifying procedures that impact the transport and fate of OC and PP to maintain the ecological health of water bodies. The fallout radionuclide (FRN), including unsupported lead-210 excess (^210^Pb_ex_) and cesium-137 (^137^Cs), can be used as geochronometers to date sediment cores and estimate changes in mass accumulation rates (MAR) over time (Appleby, 2008). The reduction rate of ^210^Pb_ex_ activity with depth establishes an age-depth relationship in the core (IAEA, 2003). While ^210^Pb_ex_ has been proven useful to reconstruct geochronology in sediments deposited over the last 100 years, due to its natural radioactive decay rate in a sediment column, an independent marker such as ^137^Cs, is needed to verify calculations resulting from ^210^Pb_ex_ activity (Foucher et al., 2021; Smith, 2001). More precisely, ^210^Pb_ex_ serves as a tool to date the entire sediment column, whereas ^137^Cs is considered to be an event marker that is only employed for particular dates to validate the ^210^Pb_ex_ dating curve. These “date stamps” are indicated by distinct peaks, e.g., that of 1963 indicating the highest fallout on the global scale and 1986 corresponding with Chernobyl incident (Northern Hemisphere). This FRN dating technique proved to be a particularly useful tool for comprehending climate change impacts (Chen et al., 2014), to determine the impacts of historical events during the previous century (Harmon et al., 2014; Sanders et al., 2006), and to indicate OC and other nutrients’ burial rates in the last 100 years (Anderson et al., 2013; Breithaupt et al., 2014; Sanders et al., 2014, 2017).

Zarivar Lake, Kurdistan Province, Iran, is a freshwater reservoir and a habitat for diverse plant and animal life (IMA, 2008), including threatened and endangered species, e.g., Oxyura leucocephala (Maroufi & Ehyaei, 2016). The soil erosion rate estimated by the ^137^Cs method in cultivated lands situated in one of Zarivar Lake’s sub-watersheds was found to be five times higher than that of the forested areas in the same watershed, at 26 Mg ha⁻^1^ year⁻^1^ (Khodadadi et al., 2024) compared to 5 Mg ha^−1^ year^−1^ (Khodadadi et al., 2023) and less than twice the country’s average of 15 Mg ha^−1^ year^−1^. One main challenge the lake encounters is the continuous input of sediments and associated nutrient/contaminants via the river network of the watershed (Sharifinia et al., 2013). Torabi Kachoosangi et al. (2020) reported the average sedimentation rate of 5.6 mm year^−1^ based on ^210^Pb_ex_ activity profile in one core retrieved from Zarivar Lake. The lake has experienced hypereutrophic conditions over the last few decades, which can accelerate the primary productivity of the lake (Ebrahimi Mohammadi et al., 2012; Hamidian & Hassanzadeh, 2013; Sharifinia et al., 2013), posing threats to aquatic life and both local inhabitants. Moreover, over the last century, the Zarivar Lake watershed has been subjected to various anthropogenic activities, including deforestation and inappropriate land-management practices alongside the implementation of conservation measures like check dams. Nevertheless, agricultural practices such as minimum tillage, contour farming, and the construction of terraces and check dams can serve as adaptive management measures to mitigate the impacts of climate change. Zarivar Lake was selected as the study site due to its high soil erosion and sedimentation rates, significant history of land-use changes, and the construction of numerous check dams in 2008–2009, providing an opportunity to analyze their hydrologic impacts. Thus, the unique characteristics of the watershed make it an ideal site for studying anthropogenic impacts under changing climate conditions. Furthermore, the region is expected to undergo substantial climate changes in the future, which is likely to influence soil erosion rates, with modifications expected in the erosive power of rainfall, soil moisture, plant biomass, litter cover, soil erodibility, and land use, especially in semi-arid areas (Nunes & Nearing, 2011). These changes, in turn, will likely impact soil erosion, the amounts of sediment entering the lakes, nutrient delivery, and internal lake production.

Zarivar Lake watershed has been the subject of a number of studies, including Ebrahimi Mohammadi et al. (2012), Hamidian and Hassanzadeh (2013), Sharifinia et al. (2013), Torabi Kachoosangi et al. (2020), and Khodadadi et al., (2023, 2024). However, to date, there has been no investigation into the accumulation rates of OC and PP in the lake, nor has the role of anthropogenic activities in upstream watersheds on MAR and nutrient burial in sediments been explored over time. In addition, despite the significant impacts of LUC on sediment and nutrient supply, as indicated by Anderson et al. (2013) and Lin et al. (2021), this effect has only been examined at a limited number of sites worldwide and thus needs further investigation. Thus, the objectives of the study were to (i) quantify land-use/land-cover (LULC) changes using aerial photos and satellite data between 1955 and 2008; (ii) estimate the sediment, organic carbon, and particulate phosphorus accumulation rates using ^210^Pb_ex_; and (iii) understand anthropogenic and climatic impacts on sediment, OC, and PP accumulation rates using LULC changes, mean annual precipitation, δ^13^C, and elemental indices over the past decades in one of sub-watersheds of Zarivar Lake.

## Materials and methods

### Study area

**Fig. 1:**
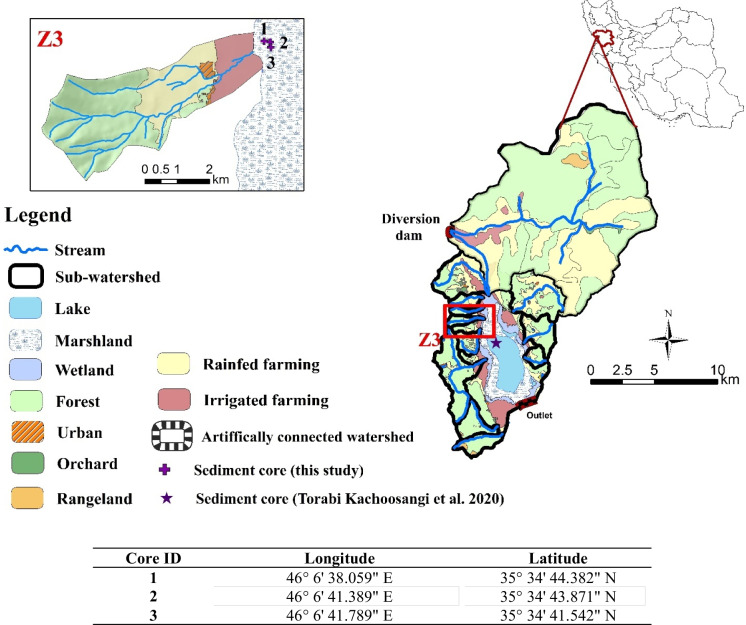
The location of Zarivar lake watershed, Z3 watershed, and the sediment cores in marshland (this study) and in lake (Torabi Kachoosangi et al., 2020) in Iran. The coordinates of the cores are also provided

Zarivar Lake (35° 33′ 15″ N and 46° 7′ 25″ E) is a natural shallow reservoir situated approximately 15 km east of Iran-Iraq border (Fig. 1). The lake, which is inhabited by aquatic plants within its shallow regions, e.g., *Typha* spp. (i.e., cattails or common bulrush) and *Phragmites* spp. (common reed), has a surface area of ca. 18.3 km^2^ and a maximum depth greater than 6 m (IMA, 2008). Most of the rivers in the lake watershed are seasonal (non-permanent). A dike was constructed at the outlet of the watershed in 1995; therefore, the lake only drains at high water levels. Additionally, in 2004, the Ghezelchesoo River was partly diverted to an artificial seasonal tributary which flows into the lake (IMA, 2008). Subsequent to the construction of the diversion dam redirecting the Ghezelchesoo River, the lake’s watershed area expanded from 107.2 to about 296.4 km^2^ (Fig. 1). The mean annual precipitation (MAP) and temperature (MAT) are at 991 mm and 12.8 °C, respectively (Iran Meteorological Organization).

The present study was conducted in an upstream sub-watershed connected to the lake in the western part of the watershed, namely, the “Z3” sub-watershed (Fig. 1), encompassing a total area of approximately 2.97 km^2^. The selection of this watershed was motivated by findings from Ebrahimi Mohammadi et al. (2012), which highlighted that the northern and western tributaries contributed significantly higher amounts of suspended sediment and nutrients to Zarivar Lake. The watershed has an average altitude of 1518 m a.s.l. (varying between 1296 and 1884 m a.s.l.) and an average slope of 31% (ranging from to 0.1 to 82%). While the parent materials in ridges consist of metamorphic rocks of complexly folded black shale and minor metamorphosed limestone and sandstones, in the lowlands, Quaternary sediments, mainly lacustrine deposits, can be found (Geological Survey and Mineral Exploration of Iran). The sub-watershed’s soils fall into the Typic Haploxeralfs and Typic Haploxerepts categories based on Soil Taxonomy (2014). In the WRB classification, they are classified as Haplic Luvisols and Haplic Cambisols (WRB, 2014). The topsoil textures are predominantly silty loam and loam with significant variation in stone content (IMA, 2008). The landscape in this region is characterized by mountains covered by oak forests, hills, and plains which are used mainly for agriculture.

The sub-watershed encompasses three distinct land-use categories, comprising deciduous oak forests and irrigated and dry-farming agricultural lands (Fig. 1). Within the irrigated zones, common crops include alfalfa, tobacco, and strawberries. Conversely, dry-farming lands feature cereal crops such as wheat, barley, lentils, and vineyards. Notably, vineyards are predominantly situated on sloped terrains. It is important to mention that, after the harvest of cereal crops, the soil surface typically remains exposed, making it vulnerable to erosion during heavy rainfall events. Generally, different soil erosion features, including sheet, rill, stream bank, and gully (only one stabilized gully was found), have been observed in the sub-watershed. However, conservation measures have been taken to reduce sediment runoff in the sub-watershed, such as terraces and check dams in the sub-watershed in 2008–2009. The sub-watershed is an ungauged watershed without streamflow data.

### Sediment core sampling and laboratory analyses

In 2016, three sediment cores (in excess of 50 cm depth) were collected from the marshland, away from the shoreline (Fig. 1) where the water depth reaches around 3 m in wet seasons, using a purpose-built gravity corer comprising stainless-steel coring tube and a transparent plexiglass tube with a 9.4 cm diameter. This setup facilitated the visual assessment of changes in sediment texture. For this study, one of the three cores (core#2 in Fig. 1) was sliced into 1-cm-depth increments, except for the upper section of the core (i.e., 0–7 cm), where dense plant residuals made it impossible. Sediment from the top 7 cm was separated from plant material and composited for further analysis. To minimize contamination and disturbance during sample collection and preparation, we followed Gibbs (2014) sampling protocols. Water samples above the sediment columns were collected and placed in dark bottles for total dissolved phosphorus (TDP; ascorbic acid blue method, Murphy & Riley, 1962) analyses. All samples were kept in an ice box (− 4 °C) during transport to avoid changes in chemical properties.

Increment sediment samples were oven-dried at 60 °C, disaggregated, and gently ground using a mortar and pestle. The samples were analyzed for ^137^Cs, ^210^Pb, and ^226^Ra by gamma spectrometry using N-type HPGe detector at the Soil Science Unit at the IAEA Seibersdorf Laboratory. Prior to analyzing ^210^Pb, samples were sealed for 1 month to achieve equilibrium between ^226^Ra and its daughter ^214^Pb. ^137^Cs, total ^210^Pb, and ^214^Pb activities were determined from the net peak areas of gamma rays at 661.6, 46.5, and 352 keV, respectively. The ^210^Pb_ex_ mass activity was calculated by subtracting ^226^Ra from the total ^210^Pb measurement. Depending on the FRN activities, counting times ranged between 12 and 24 h, to acquire a precision of 5% to a maximum of 20% at the 95% level of confidence.

The sources of buried OC can be identified using carbon stable isotope signatures, i.e., δ^13^C (e.g., Sanders et al., 2014). OC and δ^13^C were measured in 27 of 45 increment sediment samples, which were randomly selected from various depths within the core. Initially, inorganic carbonates were removed using 10% HCl. The OC content in each sample was determined as loss on ignition (LOI) by combustion of dried soil at 500 °C for 3 h, which was then multiplied by 0.47 (Perie & Ouimet, 2008) to estimate total organic carbon. The δ^13^C isotopic signature of organic carbon was measured in acidified samples using a Delta V Plus continuous flow isotope ratio mass spectrometer (CF-IRMS) linked to a Flash 2000 elemental analyzer on a MAS 200 R autosampler (Thermo-Fisher Scientific, Bremen, Germany) at Environmental Stable Isotope Laboratory, the National Institute of Water and Atmospheric Research, Wellington, New Zealand. To make δ^13^C values with different ages comparable, the Suess effect—a decrease in δ^13^C signature of atmospheric carbon dioxide (CO_2_) due to burning fossil fuels with δ^13^C depleted CO_2_ emissions (commencing in the 1700s)—correction was required (Verburg, 2007). This correction was performed using Verburg’s (2007) sixth-order polynomial equation to calculate isotopic depletion. Then, the absolute δ^13^C value (− 8.65‰) of present-day CO_2_ (the year 2016) was added to compute the change in δ^13^C isotopic values for each year of the core (Gibbs, 2014).

Sediment increment samples were also analyzed for major and minor elements using wavelength dispersive X-ray fluorescence (WD-XRF) with the OMNIAN application on an Axios Max instrument (Malvern PANalytical, Malvern, UK) at the University of Plymouth, Plymouth, UK. The procedure followed the method described by Wynants et al. (2021). Prior to pelleting, samples were homogenized by milling for 20 min at 300 rpm to reduce shadowing effects and prevent the preferential analysis of finer particles (Willis et al., 2011). Instrument calibration and drift were evaluated using internal quality control protocols with a multi-element glass sample provided by the manufacturer. Triplicates of randomly selected samples were prepared to assess the repeatability of the method. The instrument measurements were validated with stream sediment certified reference material (GBW07318, LGC, Middlesex, UK). Geochemical constituents of the samples measured were P, Na, Mg, Al, Si, S, Cl, K, Ca, Sc, Ti, V, Cr, Mn, Fe, Co, Ni, Cu, Zn, Ga, Br, Rb, Sr, Y, Zr, Nb, Sn, I, Cs, Ba, La, Ce, Nd, Hf, Ta, Hg, Pb, Th, and U.

### Core dating and MAR, OCAR, and PPAR calculation

The constant rate supply (CRS) model was applied to establish the age-depth relationship according to the ^210^Pb_ex_ depth profile (Appleby & Oldfield, 1978). The CRS model is considered to be a very practical dating approach and is widely used and validated by numerous independent approaches (Appleby, 2008; Foucher et al., 2021). The CRS model presumes that the input of ^210^Pb_ex_ to the sediment is constant throughout time (Krishnaswamy et al., 1971), which is a widely accepted assumption (e.g., Appleby & Oldfield, 1992) and that MAR can be modeled using the ^210^Pb_ex_ profile shape and radioactive decay (Appleby & Oldfield, 1978). We calculated CRS ages and MARs for each increment depth using the equations provided by Appleby (2001). MAR was reported as g cm^−2^ year^−1^ or kg m^−2^ year^−1^ and converted to sediment accumulation rate (SAR; mm year^−1^) using corresponding bulk density at each interval. Wet and dry bulk densities were calculated for sediment increments using the core radius, increment depth, and weight of each increment. For each increment depth, the mid-point of that layer was used in calculations.

OCAR and PPAR were computed by simply multiplying the total sediment mass accumulation rate at each increment depth by the respective proportion of OC or PP and expressed as g m^−2^ year^−1^ (e.g., see Waters et al., 2019). Subsequently, to evaluate significant differences between estimated accumulation rates in different sections of the core, we used a Kruskal–Wallis test followed by Dunn’s post-hoc test with Bonferroni correction in R. Significance was assessed at *p* < 0.05 level.

### Geochemical and geoaccumulation indices

Using elemental compositions, the geochemical indices were calculated to assess sediment maturity, chemical weathering, and depositional environment in different sections of the core. Two indices were applied to deduce the level of chemical maturity of the sediment column: the index of chemical maturity (ICV; see Table 1) and the SiO_2_/Al_2_O_3_ ratio. Additionally, the chemical index of alteration (CIA; refer to Table 1) was used to evaluate the extent of chemical weathering in the sediments. To understand the bottom lake conditions, i.e., oxic, sub-oxic, and anoxic, various elemental ratios such as U/Th, V/Cr, and Ni/Co were applied (Armstrong-Altrin et al., 2015; Jones & Manning, 1994).

**Table 1:** The average (range) of geochemical indices for sediment maturity, chemical weathering, and depositional environment in different sections of the Zarivar Lake core

Depositional environment	Geochemical index	Observed mean (range)				Classification	Classification criteria	Reference
		Upper (0–15 cm)	Middle (15–25 cm)		Lower (25–50 cm)			
Sediment maturity	Si_2_O/Al_2_O_3_	2.74 (2.68–2.81)	2.75 (2.65–2.97)		3.05 (2.89–3.34)	Immature	Immature < 10 Mature > 10	Bakkiaraj et al. (2010)
	ICV*	1.02 (0.94–1.24)	1.02 (0.92–1.17)		0.92 (0.83–1.08)	Immature	Immature < 2 Mature > 2	Armstrong-Altrin et al. (2015)
Chemical weathering	CIA**	69.37 (62.93–72.75)		70.04 (64.06–75.21)	73.55 (67.79–76.80)	Moderate to intense chemical weathering	Intense chemical weathering > 70	Zaid (2015)
Deposition condition	U/Th	0.30 (0.24–0.34)		0.30 (0.24–0.36)	0.34 (0.29–0.40)	Oxic	Anoxic > 1.25 Oxic < 0.75	Jones and Manning (1994)
	V/Cr	0.99 (0.96–1.00)		0.99 (0.96–1.04)	1.12 (0.98–1.32)	Oxic	Anoxic > 4.5 Oxic < 2	
	Ni/Co	4.64 (4.15–5.02)		4.70 (4.11–5.16)	5.06 (4.48–5.97)	Oxic to sub-oxic	Anoxic > 5 Oxic < 5	

*Index of chemical maturity; ICV = (Fe_2_O_3_ + K_2_O + Na_2_O + CaO + MgO + MnO + TiO_2_)/Al_2_O_3_ (Cox et al., 1995)

**The chemical index of alteration; CIA = [A_l2_O_3_/(Al_2_O_3_ + CaO + Na_2_O + K_2_O)] * 100

We also utilized the degree of contamination (Dc) and pollution load index (PLI) as key indicators to assess temporal variations in overall contamination levels as a function of depth. Initially, the contamination factor (CF) for each element was determined by dividing its concentration in the core by the corresponding average shale values (AVS; Wedepohl, 1995), which were used as the background value (El-Safa et al., 2022). The degree of contamination (Dc) was calculated as the sum of the concentration factors of pollutants in a given sample (Håkanson, 1980), while the pollution load index (PLI) was determined as the geometric mean of the concentration factors (El-Safa et al., 2022).

### Evaluating anthropogenic and climatic controls on accumulation rates over the last century

#### Quantifying land-use/land-cover changes through time

Given that LU correlates with underlying elements causing changes in accumulation rates (Ferland et al., 2014; Kastowski et al., 2011), the record was paired with LU data. Land-use and land-cover changes from 1955 to 2008 were quantified using a combination of high-resolution and medium-resolution remote sensing imagery. High-resolution black and white aerial photographs, which lack spectral data, are the only source of historical LU maps in many areas (Hudak & Wessman, 1997). Imagery used for the analysis included the following: aerial photographs which were available on 8/25/1955 and 10/11/1975; Landsat 4–5 imagery available on 8/24/1982, 7/6/1984, 7/29/1985, and 8/1/1989; and Indian Remote Sensing Satellite (IRS) panchromatic (PAN) imagery available on 6/30/2008. IRS PAN provides a spectral range of 0.50–0.75 m and a spatial resolution of 5.8 m (Patwary & Parvaiz, 2009). Initially, the aerial and IRS PAN images were georeferenced in the UTM WGS84 system. To classify satellite data as forest or non-forest classes, photo-interpreted LU features along with ground-based information were used. LU classification of both types of photos is usually limited to visual interpretation involving digitizing LU classes and estimating their areas (Hudak & Wessman, 1997). Here, to produce LU maps, visual image interpretation was used in ArcGIS 10.4.4 environment.

In addition, cloud-free scenes of Landsat Thematic Mapper (TM) images (with a spatial resolution of 30 × 30 m), taken during the summer, were chosen to best identify the spectral signatures of the various LU classes. Also, for seasonal consistency, it was attempted to select them on similar dates in different years. Applied satellite data were pre-processed, meaning they had been corrected radiometrically and geometrically. Maximum likelihood classifier (Enderle & Weih, 2005) was used to classify images. Specifically, combined supervised and unsupervised classifications were applied in ERDAS 11 to enhance the accuracy of the LU maps (Muhati et al., 2018).

To enable a more accurate understanding of the relationship between land-use change and accumulation rate, the temporal aspect of deforestation was accounted for by developing a new parameter, LUC factor. Thus, LUC factors were developed for each increment by multiplying the percentage of land-use change in that period by the corresponding average slope gradient (in percentage). This approach allowed for the consideration of the extent of deforestation on slopes, taking into account the temporal sequence of deforestation.

Furthermore, areas within the forest exhibiting sparse vegetation were identified on high-resolution images, such as aerial photographs from 1955 and 1975, as well as IRS PAN image from 2008. To accomplish this, a combination of visual image interpretation and unsupervised classifications was employed using ArcGIS 10.4.4 software.

#### Quantifying climate changes through time

MAP and MAT data were obtained from daily records of two regional meteorological stations, the local Marivan station and the more distant Sanandaj station (Iran Meteorological Organization). Meteorological data was available from the Marivan station beginning in 1992. Sanandaj station is located around 90 km from the study area and has meteorological data available from 1959 to the present (Table S1). Sanandaj experiences a semi-arid cold climate, while Marivan station has a humid cold climate.

A simple yearly correlation model was built for the annual precipitation of Marivan and Sanandaj stations prior to 1992, which was then applied to produce MAP between 1959 and 1992. A trend analysis of climatic parameters was done using the non-parametric Mann–Kendall test and the non-parametric Sen method in Sanandaj and Marivan meteorological stations (Salmi, 2002). As MAPs were only available since 1959 (depth ~ 33 cm), we assumed MAPs in previous years (*n* = 4) to be equal to the average MAP (1139.0 mm). Ultimately, Pearson’s correlation and principal component analysis (PCA) were conducted to explore potential relationships between sediment parameters, climatic and land-use change factors, and accumulation rates using R-Studio. Additionally, flood reports were collected from local and national news sources via online platforms; however, they were not used in data interpretation due to concerns about their comprehensiveness.

### Upscaling MAR and OCAR to the Zarivar Lake

Although the primary objective of this study was to investigate the anthropogenic and climatic impacts on sediment accumulation rates in a small sub-watershed (i.e., Z3) connected to Zarivar Lake, the accumulation rates from this study were also used in a two-compartment model to improve estimates for in the entire lake and inform whole-large management strategies. The estimation of whole lake MAR was done by combining observations from the core collected for this study in the marshlands with observations from a core collected in the center of the lake, as reported by Torabi Kachoosangi et al. (2020). Core locations are shown in Fig. 1. In this approach, it was assumed that the center-lake core was representative of the non-vegetated lake regions (8.8 km^2^) and that the marshland core was representative of the marshland areas (8.9 km^2^). As accumulation rates within marshlands and center lake might differ due to lower velocity and higher deposition rates as well as higher nutrient cycling in marshlands compared to the lake center.

The approximate calculations were conducted for the whole lake, including sedimentation rate, sediment production per unit area of the lake watershed, the amount of sediment stored behind the check dams of the Z3 sub-watershed over an 8-year period, and the OCAR for recent sediments. To achieve these, a method was employed to upscale the burial rates of single sample data to estimate whole lake burial rates. This method involved computing a focusing factor, as described by Engstrom (2005). The focusing factor is defined as the ratio of the observed ^210^Pb_ex_ accumulation rate in the core (F[^210^Pb_site_]) to the known atmospheric flux of ^210^Pb_ex_ in the region (F[^210^Pb_atm_]; Rippey & Douglas, 2004), which is approximately 216 Bq m^−2^ year^−1^ in the region under investigation (Khodadadi et al., 2023). The sediment production per unit area of the Zarivar Lake watershed was estimated by dividing the annual mass accumulation in both the waterbody and marshland by the total area of the watershed. Additionally, using an average net soil erosion rate and sediment production per unit area of Z3 watershed, we estimated the sediment delivery ratio (SDR) and the volume of sediment trapped behind the check dams over an 8-year period. The former was approximated by dividing the annual sediment production by the erosion amount, while the latter was determined by subtracting the difference between the erosion amount and sediment production over the 8-year period. Similarly, whole lake OCAR was computed based on surface sediments (ca. 10 years of accumulation), following the method described by Hobbs et al. (2013).

## Results

### Geochronology and accumulation rates

**Fig. 2:**
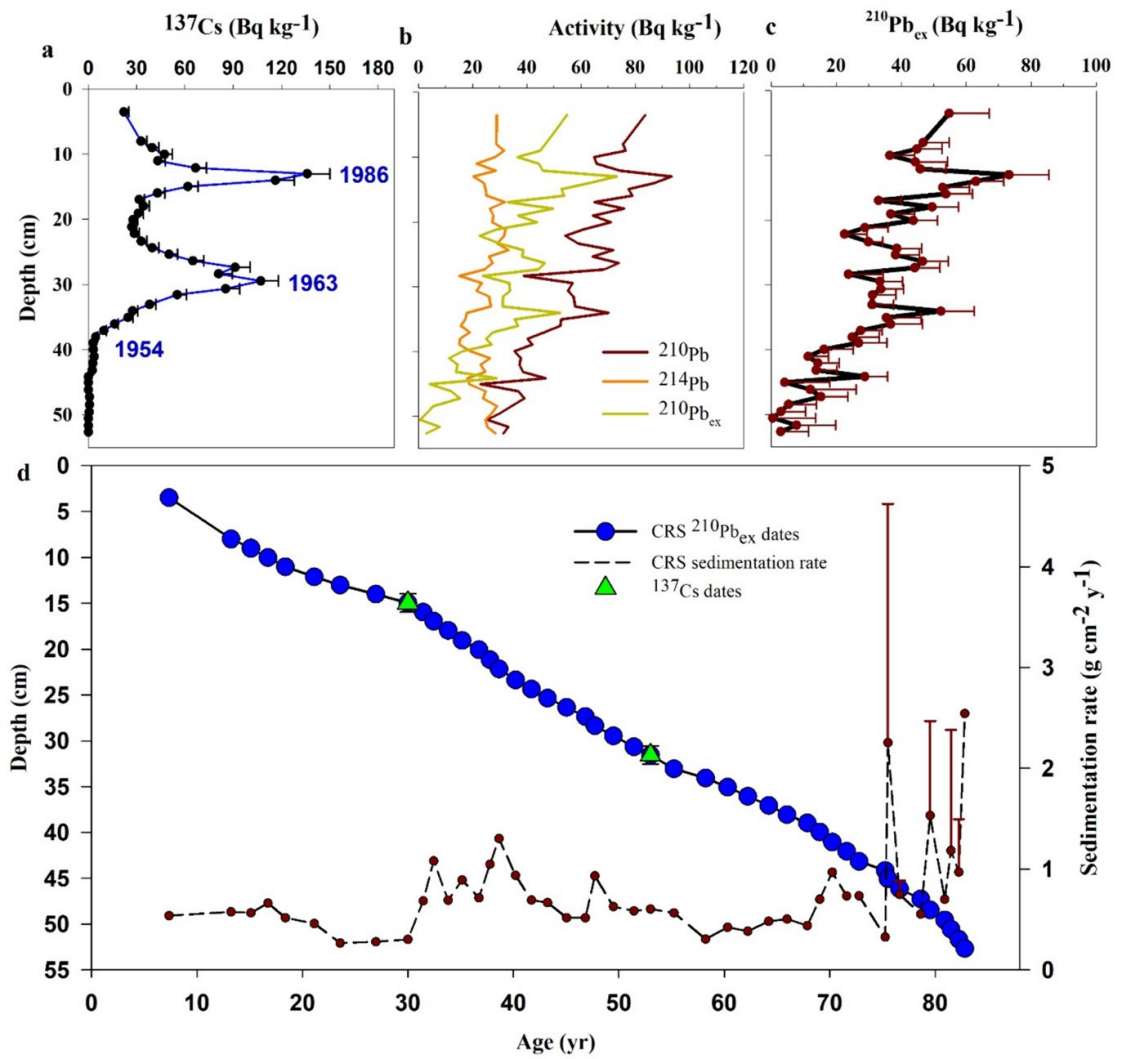
Depth profile of ^137^Cs (with analytical error bars (**a**)); ^210^Pb, ^214^Pb, and ^210^Pb_ex_ (**b**); ^210^Pb_ex_ (with analytical error bars (**c**)); and composite CRS model chronology with the 1986 and 1963 depths indicated by the ^137^Cs records (**d**) (the record was sub-divided into two sections at 14 cm, i.e., 1986). Sedimentation rates (g cm^−2^ year^−1^) and errors from the CRS model are also shown

The ^137^Cs profile indicated two distinct peaks at depths of 13 and 30 cm, corresponding to 1986 and 1963 peaks, respectively (Fig. 2a), while ^210^Pb_ex_ profile represented an extremely non-uniform depth profile. The irregular ^210^Pb_ex_ profile indicated that there may have been significant changes in the sedimentation rate over the past century. For such variable ^210^Pb_ex_ depth profile, the CRS model is normally used for the stratigraphic chronology (Fig. 2b). However, a small discrepancy between the CRS model dates and the 1986 and 1963 depths determined from the ^137^Cs records was observed. Therefore, an approach, known as composite modeling, was applied to calculate the corrected ^210^Pb_ex_ dates for the Zarivar Lake core (Fig. 2c; for more details on composite models, see Appleby, 2008). This was in accordance with elsewhere, e.g., in Blelham Tarn Lake, UK (Appleby, 2001, 2008) and Żabińskie Lake, Poland (Tylmann et al., 2016). This can be due to various causes such as flood events, sediment slumps, turbidity currents, and major land-use and land-management changes (Appleby, 2001). Here, the most probable reason for this discrepancy was the substantial reduction of sediment supply following the construction of the check dams in the river network since 2008. In general, in the case of an irregular ^210^Pb_ex_ record, the CRS model is normally applied in a piecewise way in various zones of the core in which the chronostratigraphic dates are deployed as reference points, an approach that yields corrected ^210^Pb_ex_ dates (Appleby, 2008; Tylmann et al., 2016). Thus, composite CRS model was applied to calculate the corrected ^210^Pb_ex_ dates and MAR. Based on this method, we sub-divided the record into two sections, i.e., below 13 cm (pre-1986) and above 13 cm (post-1986), and calculated age and MAR for all intervals in each section.

**Fig. 3:**
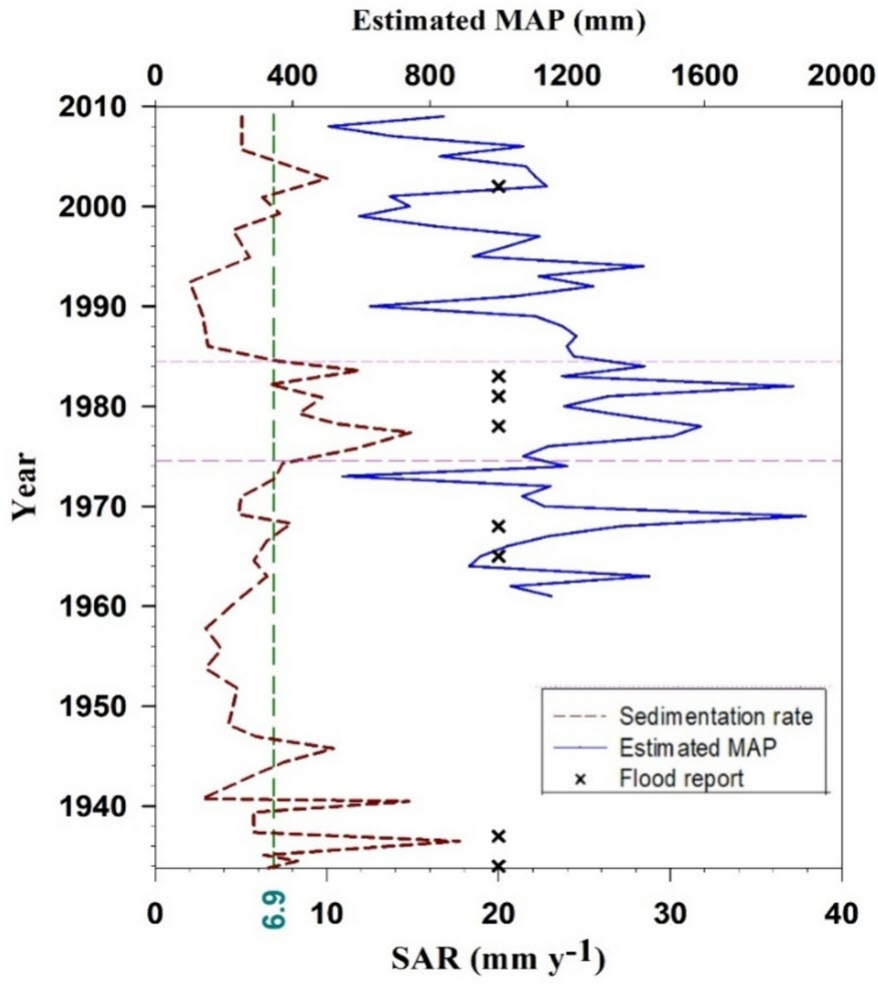
Estimated SARs (mm year^−1^) using composite CRS model in the Zarivar Lake. Dashed horizontal lines bracket a period with high SARs. Average SAR (dashed green line), estimated MAPs (solid blue line), and reported floods in the region and country ( ×) through time are shown as well. Note: Flood reports were collected from local and national news sources through online platforms and were not used in the interpretation of the data

Unusually high SARs were observed at the bottom of the core (> 50 cm; Figs. 2b and 3), which could be attributed to reaching the end of the ^210^Pb_ex_ profile, uncertainty of ^210^Pb measurement, or rainfalls with a 100-year return period. As calculating the ages in depth with certainty in depths > 50 cm proved challenging without applying independent tracers, e.g., ^14^C (Tylmann et al., 2016), this section was excluded from further calculations.

**Fig. 4:**
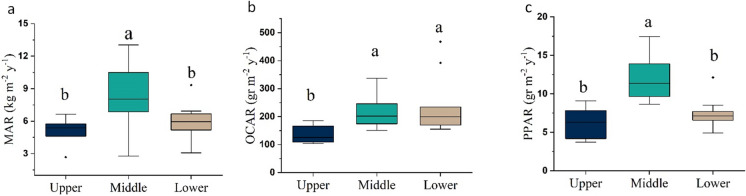
The range of MAR (**a**), OCAR (**b**), and PPAR (**c**) in upper (0–15 cm), middle (15–25 cm), lower (25–50 cm) sections of the sediment core. The Kruskal–Wallis test indicated a significant difference among the groups for MAR (*χ*² (chi-square) = 6.70, *p* = 0.0350), OCAR (*χ*² = 10.01, *p* = 0.0067), and PPAR (*χ*² = 15.58,* p* = 0.0004). Pairwise comparisons were conducted using Dunn’s post hoc test with Bonferroni correction. Different letters indicate significant differences at the *p*-level < 0.05

Sedimentation rate varied considerably through time (Fig. 3), and a period of significantly higher-than-average SARs was identified. Subsequently, we sub-divided the core into three sections based on SAR, i.e., 0–15 cm (upper), 15–25 cm (middle), and > 25 cm (lower). ANOVA showed a significant difference in MAR across sections (*p* < 0.01; Fig. 4a). The mean dry bulk density stood at 0.99 ± 0.19 g cm^−3^, being the highest in the upper 15 cm (1.08 ± 0.26 g cm^−3^), mid-section at 1.04 ± 0.09 g cm^−3^, and slightly lower at 0.91 ± 0.18 g cm^−3^ in the bottom section. The average CRS-derived MAR was 6498 ± 2475 g m^−2^ year^−1^ (SAR = 6.9 mm year^−1^).

**Fig. 5:**
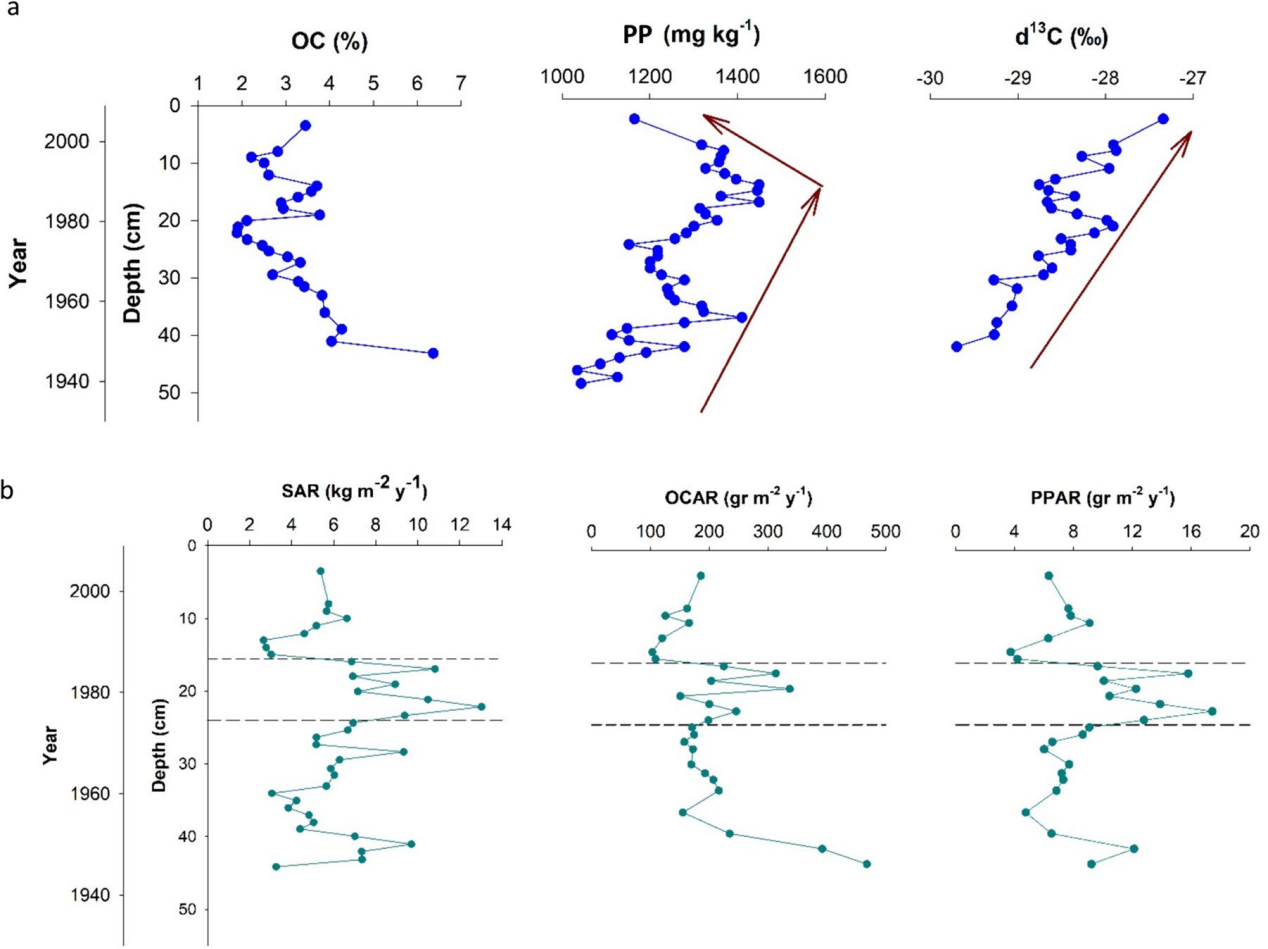
Depth profile of various parameters in the Zarivar Lake, including total organic carbon (OC; %), particulate phosphorus (PP; mg kg^−1^), and δ^13^C (Suess effect corrected; ‰) contents (**a**) and depth profiles of mass accumulation rate (MAR; kg m^−2^ year^−1^), organic carbon accumulation rate (OCAR; g m^−2^ year^−1^), and particulate phosphorus accumulation rate (PPAR; g m^−2^ year^−1^) based on the composite ^210^Pb_ex_-CRS model (**b**). Brown arrows show the general trend of the parameters with depth. Dashed lines bracket a period with distinct high MARs

The mean OC content and PP for all increments were 3.2 ± 0.9% and 1035 ± 108 mg kg^−1^, respectively (Fig. 5a). OCAR and PPAR estimates were calculated based on MAR derived from ^210^Pb_ex_-composite CRS model and representing an 80-year time span. Mean OCAR and PPAR in all intervals stood at 205 ± 85 g m^−2^ year^−1^ and 8.9 ± 3.3 g m^−2^ year^−1^, respectively (Fig. 5b). OCAR was significantly lower in the upper zone of the core (Fig. 4b). The PPAR in the middle section was significantly higher than in the two other sections (Fig. 4c).

### Elemental geochemistry

The ICV ranged from 0.83 to 1.24 and the SiO_2_/Al_2_O_3_ ratio varied between 2.65 and 3.34, highlighting a low level of compositional maturity across various sections of the core (Table 1). The CIA values of 69.37, 70.04, and 73.55 for the upper, middle, and lower core sections, respectively, revealed moderate weathering in the two top sections but an intense weathering condition for sediment in the bottom section (Table 1). Zarivar Lake sediments demonstrated low U/Th varying from 0.24 to 0.40 and V/Cr ranging from 0.96 to 1.23, suggesting an oxic depositional environment (Jones & Manning, 1994; Table 1). Ni/Co ratios were at 4.64, 4.70, and 5.06 in the upper, middle, and lower sections, respectively, indicating an oxic depositional environment in the upper parts but a sub-oxic one in the lower zone.

The multi-element pollution indices, i.e., Dc and PLI, were computed based on the concentrations of Al, Cu, Co, Cr, Fe, Mn, Ni, P, V, and Zn (Table S2). Dc values ranged from 11.5 to 14.1, indicating a moderate contamination level (8 < Dc < 16; Håkanson, 1980), while PLI values varied between 1.0 and 1.3 in the samples, placing them in the polluted group (PLI > 1; Håkanson, 1980). Both indices exhibited significant differences across different core sections, with the lowest values observed at the bottom core section, followed by the top section. This suggests that elevated levels of contamination can enter the lake at higher MARs. This is also reflected in the positive significant correlations (at *p* > 0.05) observed between Dc and PLI and MAR (Fig. S1).

### Quantifying land-use/land-cover change over time

**Fig. 6:**
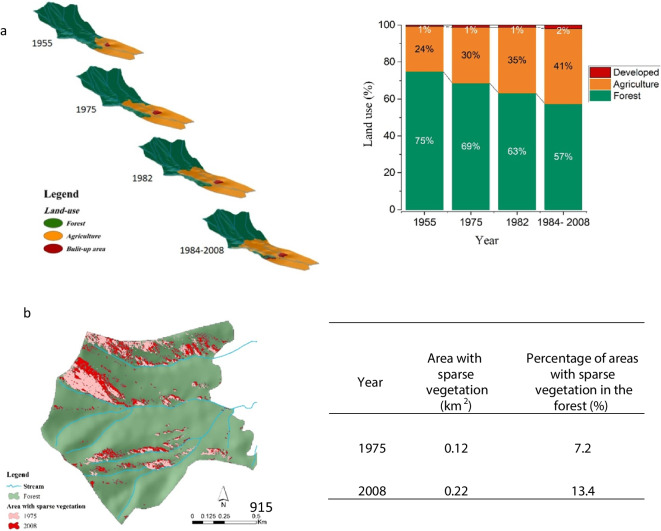
3D images of temporal sequence land-use classifications and the percentage of forest converted to agriculture in the watershed between 1955 and 2008 (**a**) and expansion of areas with sparse vegetation in forest in 1975 (pink) and 2008 (red) (**b**). Note that investigating satellite data for 1984, 1985, 1989, and 2008 revealed no significant changes in land use within the watershed

Aerial photos and satellite data illustrated that compared to 1955, the net forest cover had declined by 6 and 18% in 1975 and 1984, respectively, decreasing from 75% of the watershed area 1955 to 57% in 1984 (Fig. 6a). It is notable that 12% of forests been cleared between 1975 and 1984 and in addition, based on the digital elevation model (DEM), after 1975, deforestation took place on steeper slopes (< 10% vs 20–30%) (Fig. 6a). In contrast, from 1984 to 2008, major LUCs did not occur except for the quite limited expansion of developed areas.

In addition, the visual interpretation of high-resolution data, i.e., the aerial photo from 1975 and the 2008 IRS PAN imagery, showed that the areas with sparse vegetation had almost doubled from 0.12 to 0.22 km^2^ during the period between 1975 and 2008, with most of these areas being situated on southern slopes (Fig. 6b). This could be due to both human interferences like grazing and wood harvest (cutting branches from trees) as well as climate change.

### Quantifying climate change over time

The trend analysis of climatic parameters using the Mann–Kendall test and Sen’s slope estimates (Salmi, 2002) showed a clear decreasing trend in MAP in the study area (1959–2017; Fig. S2). However, despite an upward trend in average minimum and maximum temperature in the regional data (Sanandaj station), there was no trend detected in the watershed temperature data (Marivan station; Fig. S2). This could be due to the presence of the lake preventing temperature variabilities between 1992 and 2017. Consequently, the impacts of temperature on accumulation rates were considered to be marginal in the region.

## Discussion

### Interpreting erosional history through changes of sediment geochemistry indices through time

Elemental geochemistry can help assess sediment maturity, chemical weathering, and depositional environments in a core. A matured sediment is composed mainly of stable minerals such as quartz, so it is unlikely to undergo further weathering processes due to the absence of unstable minerals (Pettijohn, 1975). Based on the ICV (> 0.83) and the SiO_2_/Al_2_O_3_ ratio (> 2.65) values, a low compositional maturity was proved in the core (Table 1). High ratios of SiO_2_/Al_2_O_3_ usually correspond with high quartz content, indicating chemically mature sediments (Mahamuda, 2015). Owing to the dominance of expandable clays (smectite group or 2:1) measured by semi-quantitative X-ray diffraction (XRD) of the clay fraction of soils in the region (particles < 2 μm; Khodadadi et al., 2024), such SiO_2_/Al_2_O_3_ ratios were expected in the sediments (particles < 63 μm). The CIA values revealed moderate weathering in the two top sections but an intense weathering condition for the bottom section (Table 1). This suggests older contributions were from forest topsoil and more recent inputs eroded from agriculture or deeper in the soil profile, e.g., incisions.

### Anthropogenic and climatic controls on SAR

The average SAR of the lake was estimated to be at 6.90 ± 3.45 mm year^−1^, varying between 2.02 and 17.73 mm year^−1^ (Fig. 3). This value was close to the average sedimentation rate of 5.6 mm year^−1^ documented by Torabi Kachoosangi et al., (2020; Fig. 1), but the small difference can be due to differences in sampling locations within Zarivar Lake. Although sedimentation rates in different lakes can be influenced by many factors, making comparisons challenging, we present findings from several studies conducted in similar environmental settings. For instance, Morellón et al. (2011) studied climate changes and human activities recorded in the sediments of a shallow lake, draining a small catchment of 1.07 km^2^, in Spain. The catchment’s lowlands are under farming lands dedicated to barley cultivation, while higher elevation areas are mostly occupied by scrublands and oak forests. Two short cores were collected and sub-sampled every 1 cm for ^137^Cs and ^210^Pb dating. The sedimentation rate over the past 50 years was estimated at 3.41 mm year^−1^, which is lower than our estimated value. This difference can be attributed to the larger area and unsustainable management practices in Z3 sub-watershed. In a study, Valero-Garcés et al. (2006) examined human impact on a Mediterranean lake in Spain with a watershed of 900 km^2^ and a MAP of 538 mm. Soil erosion intensified in the mid-twentieth century due to farm machinery introduction. In the early 1980s, the lake became a protected area with restricted agricultural practices and implemented conservation measures. Two sediment cores were retrieved for ^210^Pb and ^137^Cs dating, revealing a mean sedimentation rate of 15 mm year⁻^1^ over the past half-century. This rate was substantially higher than our 80-year average, which may be due to the extremely large area of the watershed compared to Z3. In another study, Barreiro-Lostres et al. (2015) investigated human-climate interactions on SAR in a Mediterranean mountain lake in Spain, with a basin area of 11 km^2^ and a MAP of 956 mm. The uplands are dominated by pine trees, while cereal fields are found at lower elevations. Using ^210^Pb and ^137^Cs dating, they determined a SAR of 10 mm year⁻^1^ for the past 60 years. Although higher than our predicted value, it is notable that this sediment comes from a watershed four times larger than our study site, indicating unsustainable management practices in our studied watershed.

To gain a comprehensive understanding of the anthropogenic and climatic impacts on SAR, two specific time periods, 1975–1988 and 2008–2016, characterized by the highest levels of human interference in the watershed, were examined. Between 2008 and 2016, the amount of sediment entering the lake substantially reduced. It can be inferred that check dams managed to store almost all mobilized sediments in the watershed, demonstrating the effectiveness of the measures taken. This is supported by field observations of substantial sediment trapped behind the numerous check dams, which resulted in the majority of them being almost full of sediment in merely 8 years (see Fig. S4). Khodadadi et al. (2022) evaluated the main sediment sources in the sub-watershed before (1995–2009) and after (2009–2016) introducing the check dams to the sub-watershed using geochemical fingerprints. The results showed a significant shift in the main sediment sources from channel banks to uncultivated subsoil following the establishment of the check dams, which verifies their effectiveness in stream bank stabilization, at least for the duration of their infill stage. The efficiency of check dams in decreasing catchment sediment yield has been confirmed elsewhere (e.g., Boix-Fayos et al., 2008; Tiessen et al., 2011). However, their impact is known to be short-lived (Tiessen et al., 2011), and further investigation on their effectiveness is thus recommended in the sub-watershed in the near future, particularly due to the observed dramatic decline in storage capacity.

Between the late 1970s and early 1980s, the MAR was significantly higher than its average at 7.94 ± 3.12 kg m^−2^ year^−1^ (Fig. 4a). Scrutinizing LULC changes through time (Fig. 6a) and estimated MAPs (Fig. 3) in the watershed suggested that this may have been the result of combined human activities and climatic events (Figs. 4a and 5a). As previously noted, most of the LUCs (12%) in the watershed occurred between 1975 and 1984. Extensive deforestation after Iran’s revolution (1979) and the war between Iraq and Iran (1980–1988) were likely to have played a role. More precisely, between 1978 and 1980, the area experienced extensive deforestation subsequent to the revolution and probably caused by lack of law reinforcement, coinciding with higher MAPs (more than double). In the period between 1981 and 1983, limited military activities began in mountainous areas (building ditches) and the population started to increase (from around 350 persons in 1976 to more than 750 persons in 1986; Statistical Center of Iran), happening along with the higher MAPs. Together, these factors seem to have caused excessive amounts of sediment to enter the lake.

**Fig. 7:**
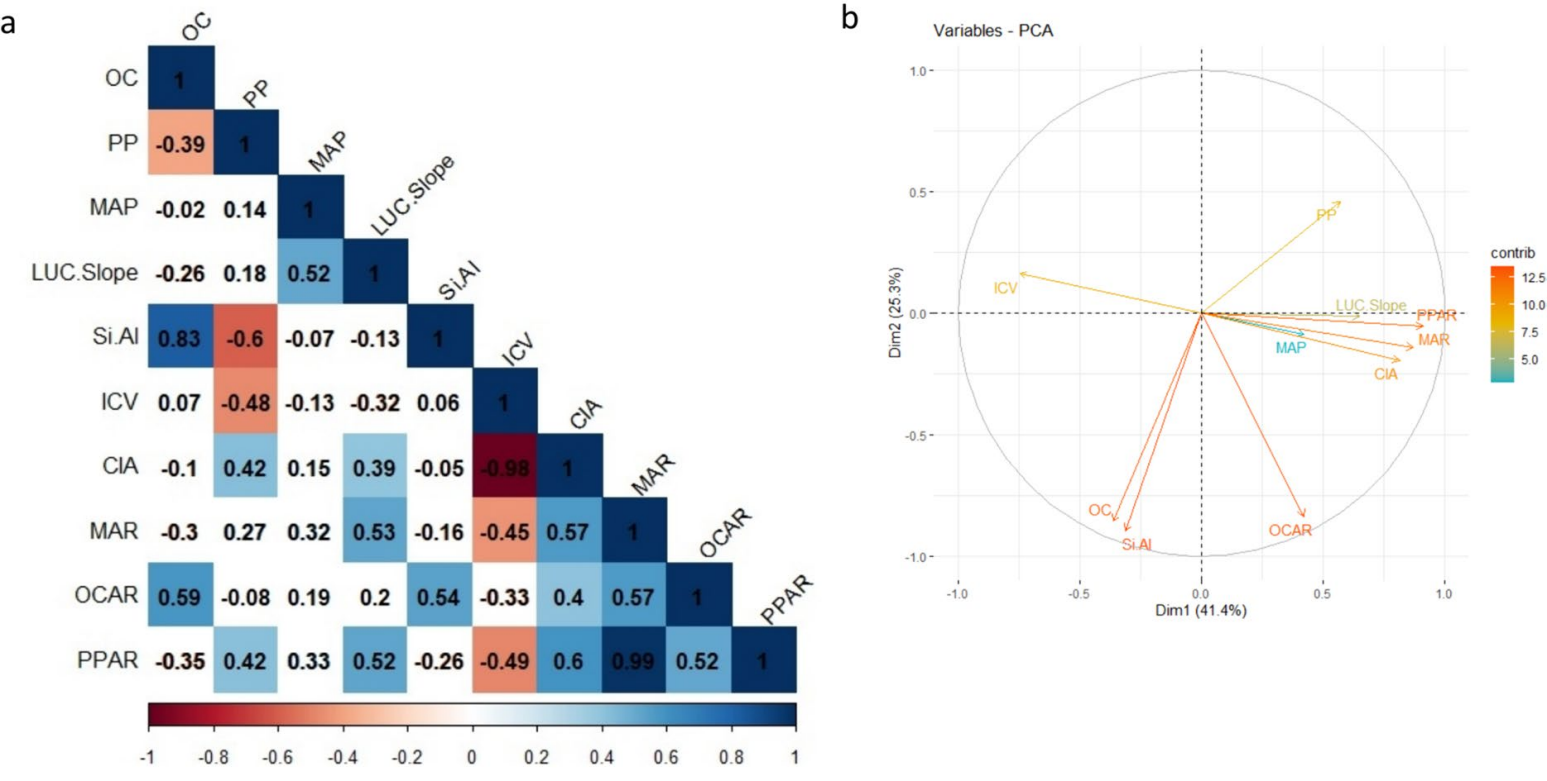
Pearson correlation coefficient (**a**) and PCA1 and PCA2 (**b**) of sediment properties, MAP, land-use change factor (percentage of land-use change multiplied by its average slope), and accumulation rates up to 40 cm (*n* = 27). Non-colored cells in 7a indicate that correlation was insignificant at *p* < 0.05. Note: Si.Al = SiO_2_/Al_2_O_3_ ratio and LUC.Slope = LUC factor

To elucidate the impact of LUC and MAP on SAR, correlation coefficients were established between accumulation rates and sediment properties, MAP, and land-use change factor up to the depth of 40 cm, which corresponds to the maximum depth of measurement for OC (1955; Fig. 7a). While a significant correlation was observed between SAR and LUC factors (*r* = 0.53, *p* < 0.01), there was no significant relationship between SAR and MAP (*r* = 0.32). This suggests that human activities had more pronounced effects on SAR, while climatic factors amplified anthropogenic impacts. Notable is that in the absence of extensive LUCs but high amounts of estimated MAP, similar to the events occurring in 1968, SAR increased only slightly (Fig. 3). All in all, it can be claimed that human-induced activities have resulted in varying levels of SAR, ranging from values that are twice the average rates to insignificant amounts. Olley and Wasson (2003) showed how small climate variabilities and a range of human activities combined had impacted sediment flux in a watershed in Australia over a period of 180 years, resulting in a non-linear relationship between SAR and rainfall, runoff, and sediment transport capacity. Having reviewed numerous case studies, Foster et al. (2003) recognized the history of LUCs as the primary element influencing sediment flux in the USA. It should be noted that accumulation rate and flux are not the same: the former refers to the amount of materials or nutrients that build up in a specific area over time, while the latter refers to the rate at which materials or nutrients are transferred into or out of a system.

Although the accumulation rates were highly sensitive to both anthropogenic and climatic factors that occurred in the sub-watershed, some of the changes in the lake seem not to impact accretion rates. For instance, the diversion dam constructed on a river, which drains agricultural lands containing high amounts of sediment and nutrients, did not exert any considerable influence on accumulation rates in 2004 or thereabouts. This may be due to the core location, which was close to the studied sub-watershed in the west of the lake.

### Anthropogenic and climatic controls on OCAR and PPAR

The mean OCAR in the entire core was estimated to be 205 ± 85 g m^−2^ year^−1^, which is 20-fold greater than the reported global means of 4 to 14 g m^−2^ year^−1^ (Cole et al., 2007; Tranvik et al., 2009). However, agriculturally impacted lakes, like Zarivar Lake, are subject to receiving high amounts of nutrients, which in turn stimulates autochthonous primary production, as well as sediments, caused by higher susceptibility to erosion of tilled soils. Consequently, the OCAR in these lakes is likely to be higher than lakes in catchments with less agricultural activity (Downing et al., 2008). There was previously no report on OCARs for Zarivar Lake; thus, we compared our value with a study in a similar environmental setting. Dominik et al. (2023) studied the organic carbon burial rate in a Mediterranean lagoon in Italy. Using four cores and the ^210^Pb dating technique, they estimated a mean SAR of 3.6 mm year⁻^1^ and OCAR of 49 g m⁻^2^ year⁻^1^. While the SAR is somewhat close to our estimated average, the OCAR is substantially smaller than our predicted value, highlighting unsustainable management in our study area.

The depth profile of the OC indicates that, except for the lowest increment depth (OC = 6.4%), OC fluctuated between 1.9 and 4.3% in the rest of the depth intervals. OCAR at upper, middle, and lower sections were 140 ± 30, 220 ± 60, and 240 ± 100 g m^−2^ year^−1^, respectively (Fig. 5b). Torabi Kachoosangi et al. (2020) also reported an increasing OC trend with depth, particularly at depths > 26 cm, in a core in the center of Zarivar Lake, which is consistent with our findings. Several factors can cause such an increasing trend in OC with depth. Firstly, the coefficient of variation (CV) of OCAR in the upper section of the core was significantly lower than in the other zones. LULC changes greatly affect OC export from terrestrial ecosystems (Smith et al., 1999), meaning that OCAR is likely to be relatively constant when a significant LULC change has not occurred (Anderson et al., 2009; Kortelainen et al., 2004). This finding is consistent with our results, as the extent of LUCs was minimal after 1984. Secondly, as U/Th and V/Cr ratios showed that the record was in oxic conditions in all intervals (Table 1), a dramatic reduction in OC in the core can be a reflection of lower OC contents of the sources, i.e., soils of the region. To illustrate, Khodadadi et al. (2023) reported that following deforestation (in 1981) on a hillslope in the sub-watershed, soil loss increased by five times, resulting in a 10-cm net soil loss, and soil organic carbon stock (SOCS) decreased by 30% (14 Mg C ha^−1^) within a course of 35 years. As such, 8 Mg C ha^−1^ of SOCS was removed by erosion, and the remaining seemed to be lost via emissions over the period in question. Thus, progressive conversion of oak forests to cropland along with an increase in areas with sparse vegetation (Fig. 7b) typically characterized by a lower OC content (Fig. S3) might have reduced OC content in the topsoils of the region through time. Together, it seemed that a decline in forest area and density, climate change (lower MAPs), and accelerated soil erosion had led to a lower OC in soils of the region since the 1980s. This hypothesis, however, may need further testing to ensure that sub-oxic conditions in lower layers, shown by the other geochemical index, i.e., Ni/Co ratio, have not impacted the organic material decomposition since it is known that organic material in anaerobic sediments tends to decompose more slowly (Dong et al., 2012; Ferland et al., 2014).

Another reason for lower OC content and decreases in OCARs in younger sediments could be the result of a change in OC sources through time, e.g., probably from forests with high OC to mainly subsoil sources with lower OC (Fig. S3). These changes of sediment sources over time were also inferred by a positive significant correlation coefficient between CIA and SiO_2_/Al_2_O_3_ ratio and OCAR (Fig. 7a), implying a change in sediment source over time from more mature (under forest) to younger sources (under cultivation). Furthermore, Lin et al. (2021) also found a notable decline in OCAR after the 1980s. This decrease was hypothesized to have been caused by increased OM decomposition and OC mineralization in the water column induced by increasing temperatures and lake-water thermal stratification. Although here, the temperature change was assumed to be insignificant over recent decades, it might have played a role as well.

While SAR and PPAR showed a significant correlation with LUC factors but an insignificant one with MAP, OCAR displayed non-significant relationships with both parameters (Fig. 7a), indicating that controlling factors of the SAR and PPAR drastically differ from those of OCAR (Fig. 7b). Nonetheless, many studies on lacustrine OC burial rates have also recognized LU practices as the primary contributor to observed patterns (Einola et al., 2011; Ferland et al., 2014; Kastowski et al., 2011; Kortelainen et al., 2004; Sanders et al., 2017). Anderson et al. (2013) emphasized the role of anthropogenic activities on OCAR in lake sediments, highlighting their importance over climatic factors. However, Kortelainen et al. (2013) claimed that climate was a more important element impacting OC in aquatic systems rather than land use. Here, it seems that OCAR is impacted by LUC and MAP factors to some extent (*r* ~ 0.2); however, other factors may play a role as well, e.g., change in OC sources and their OC content over time.

PPAR followed the same general pattern of the MAR with depth (Fig. 5b); i.e., PPAR was significantly higher in the mid-section, at 12.0 ± 2.8 g m^−2^ year^−1^, than in the upper and lower sections, at 6.4 ± 1.8 and 7.4 ± 1.9 g m^−2^ year^−1^, respectively (Fig. 5c). Significant correlations were observed between PPAR and MAR (0.99) as well as between PPAR and OCAR (0.52), the first of which considerably exceeds the second correlation coefficient. This implies that the process impacted inorganic particulate matter and PP transport and fate may have been similar, whereas some other processes may have impacted OCAR in the sediment core. However, it is important to note that the investigation of these processes and their changes over time is beyond the scope of this study.

There are distinctive isotope signatures in C_3_ vs. C_4_ plants (i.e., cool weather grasses, trees, and shrubs vs warm weather grasses, cereals, and crops) owing to a marked difference in ^13^C discrimination in their photosynthetic pathways (Farquhar et al., 1989). C_3_ and C_4_ plants and algae have a mean δ^13^C value of − 27‰ (− 32 to − 20‰), − 12‰ (− 17 to − 9‰), and − 22‰ (− 30 to − 20 ‰; France, 1996), respectively. The mean δ^13^C for all intervals was − 28.53 ± 0.52 ‰, and there was an incremental decrease with depth (Fig. 6a), signifying that C_3_ plants (e.g., forest) have covered the region and no abrupt ecological shifts (e.g., C_3_ to C_4_) have occurred. Sanders et al., (2014, 2017) reported a gradual decrease in δ^13^C with depth in mangrove sediments, which related to a growth in algal production. The TDP measurements at two points in the lake at 1.21 and 1.02 mg l^−1^ were in line with reported values by Ebrahimi Mohammadi et al. (2012). Thus, the lake had eutrophic conditions over the last few decades, leading to an acceleration in its primary productivity. This often includes higher rates of algal growth. Therefore, here, also higher values of δ^13^C in younger sediments may also have been caused by algal growth in response to larger amounts of nutrients entering the lake. Another possible explanation for SOC δ^13^C variations with depth could be that the vegetation found in the past might have been slightly different from existing flora (Mermut & Acton, 1984). Overall, δ^13^C imposes some limitations to reliably describing OC dynamics in ecosystems not having experienced a shift in vegetation from C_3_ to C_4_ plants (Trumbore, 2009).

### Estimating whole-lake accumulation rates

To assess the average whole-lake sedimentation rates, the focusing factor was calculated using the observed ^210^Pb_ex_ accumulation rate in the core to the known atmospheric flux of ^210^Pb_ex_ in the region. The sediment focusing factors were at ca. 1.0 and 1.2 for central (Torabi Kachoosangi et al., 2020) and marshland (this study) cores, respectively. The values were in agreement with values reported by Rippey and Douglas (2004) for lake sediments in the UK and Ireland, with focusing factors varying from 0.37 to 7.77. The whole-lake average sedimentation rate was estimated at approximately 7 mm year^−1^ and the annual sedimentation amount was 100 Gg year^−1^. The amount of sediment production per unit area of the Zarivar Lake watershed (296.4 km^2^) was computed to be approximately 3.4 Mg ha^−1^ year^−1^. Further, using the two cores, the average whole lake OCAR for near-surface sediments was estimated at 300 g m^−2^ year^−1^. From this, an estimated 5 Gg OC year^−1^ was accumulated in the lake during the past 10 years.

As for the Z3 sub-watershed, using an average net soil erosion rate of 12.6 Mg ha^−1^ year^−1^ (unpublished data), the sub-watershed area, and the sediment production per unit area of the Zarivar Lake watershed (3.4 Mg ha^−1^ year^−1^), the SDR of the sub-watershed would be around 27%. The magnitude of the trapped sediments behind the check dams during 8 years was estimated to be 8 Gg. This calculation highlights the effectiveness of check dams at this site in reducing sediment load to the lake. Overall, these estimations can provide valuable insights which could inform lake management strategies.

However, several limitations should be acknowledged in our study. To begin with, while upper increments could be precisely dated using ^210^Pb_ex_, dating bottom core increments requires ^14^C measurements. Furthermore, the study could gain insight from investigating the impacts of shifts in sediment sources over time to elucidate variations in MAR, OCAR, and PPAR. Moreover, the study could benefit from additional measurements, such as N, δ^15^N, and TP, to further illuminate the eutrophic condition of the lake over the past decades (Zan et al., 2012). Additionally, although we calculated rough estimates of entire lake accretions, enhancing the precision of sedimentation estimations across the entire lake watershed would require collecting more cores from both the marshlands and the lake. Lastly, applying our methodology to broader spatial scales can help evaluate the impacts of land-use and climate changes in each sub-watershed and across the entire lake watershed, contributing to a more holistic understanding of the lake ecosystem dynamics.

## Conclusion

Over the last century, Zarivar Lake has been subjected to anthropogenic interferences, including intensive LUCs and the introduction of some soil conservation practices. To understand the extent of the effects that these human activities and climate change on the magnitude of sediment, OC, and nutrients accretions through time, a short sediment core was collected within the lake. LUCs were quantified using aerial photos in 1955 and 1975; Landsat 4–5 in 1982, 1984, 1985, and 1989; and IRS PAN in 2008. This study revealed that MAR, OCAR, and PPAR during the late 1970s and early 1980s were significantly higher than average, which is thought to result from massive human interferences cooccurring with extreme climatic events. In contrast, human intervention in the form of check dams proved effective conservation measures, storing almost all sediment in the watershed between 2008 and 2016.

Together, these results illustrate that human activities caused accumulation rates to fluctuate from substantial amounts, with values twice the averages, to insignificant amounts following the installation of check dams, during the studied time window. Differentiating anthropogenic from climatic effects can be immensely complex at the basin scale over historical times, particularly in watersheds subjected to intense human interferences, and was not entirely possible in this study. However, the results suggested that human activities had more pronounced effects on MAR, OCAR, and PPAR while climatic factors amplified anthropogenic impacts. Overall, our study showed that applying a composite CRS model is recommended when the record experiences a hiatus in sedimentation for a while. The findings clearly demonstrated that land-use changes on steep slopes are responsible for the elevated accumulation rates of sediment, OC, and PP in lakes, particularly when these changes coincide with extreme weather events. Furthermore, while land-use and climate changes can accelerate the influx of fine sediments and associated nutrients into inland lakes, watershed conservation practices could effectively reduce the supply of fine particles. Our findings clearly demonstrated that applied conservation measures were able to mitigate the adverse impacts of climate variabilities over the past decades, i.e., the decreasing trend of MAP. However, it is important to note that check dams are considered short-term conservation measures. This means that the lake’s future accumulation rates might fluctuate significantly, particularly if new conversion measures are not placed and LUC, soil erosion, and lake eutrophication are not regulated. Therefore, it is imperative for local policymakers to implement additional check dams in the Z3 sub-watershed. These findings also provide a basis for global policymakers to enforce restrictions on deforestation on steep slopes and monitor the long-term effectiveness of implemented conservation measures.

The applied methodology has proven effective in evaluating the impacts of LUC, climate variability, and the effectiveness of conservation measures in ungauged watersheds linked to reservoirs. However, further research is essential to fully understand the impacts of LUC and the effectiveness of conservation methods in inland lakes under extreme weather events and prolonged climate shifts to ensure the long-term health of water bodies.

## Supplementary Information

Below is the link to the electronic supplementary material.

Supplementary file1 (DOCX 2.05 MB)

## Data Availability

Data availability The datasets used and analyzed during the current study are available from the authors upon reasonable request.
