# Supplementary material for: Anthropogenic and climatic impacts on historic sediment, carbon, and phosphorus accumulation rates using 210Pbex and 137Cs in a sub-watershed linked to Zarivar Lake, Iran
Source: Environ Monit Assess. 2024 Sep 4;196(10):887. doi: 10.1007/s10661-024-13048-5 (PMC11374916; doi:10.1007/s10661-024-13048-5)
Supplement: Supplementary file 1 — Supplementary file1 (DOCX 2.05 MB) [file 10661_2024_13048_MOESM1_ESM.docx]

**Supplementary Information:**

**Table S1** The characteristics of Marivan and Sanandaj meteorology stations.

| **Station** | **Elevation (m)** | **Mean annual precipitation (mm)** | **Temperature** | | | **Climate** | **Distance to study site (km)** | **Data availability** |
| --- | --- | --- | --- | --- | --- | --- | --- | --- |
|  |  |  | **Min**  **(ºC)** | **Max**  **(ºC)** | **Mean**  **(ºC)** |  |  |  |
| **Sanandaj** | 1373 | 458.4 | -5.4 | 36.8 | 13.4 | Semi-arid cold | 90 | 1959-2017 |
| **Marivan** | 1287 | 991.2 | -4.6 | 35.4 | 12.8 | Humid cold | >8 | 1992-2017 |

**Table. S2**. Elemental concentrations (mg kg^-1^), and PLI and Dc indices at different core sections. Different letters indicate significant differences at the p-level < 0.05 up to 45 cm (n=36).

| **Parameter** | **Core sections** | | |
| --- | --- | --- | --- |
|  | **Upper** | **Middle** | **Lower** |
| **Na** | 5058.94±383.06^a^ | 4777.99±317.33^b^ | 4664.12±176.20^b^ |
| **Mg** | 15562.43±757.74^c^ | 16790.2±241.36^a^ | 16314.00±434.74^b^ |
| **Al** | 102008.67±6819.32^a^ | 105181.18±1250.92^a^ | 98405.20±3148.60^b^ |
| **Si** | 246009.19±14125.63^b^ | 249575.82±3792.63^ab^ | 256270.82±8224.06^a^ |
| **P** | 1346.43±77.98^a^ | 1324.7±87.45^a^ | 1241.80±72.12^b^ |
| **S** | 849.68±445.96^a^ | 570.53±125.02^b^ | 796.00±262.38^ab^ |
| **Cl** | 212.5±44.72^a^ | 145.00±19.00^b^ | 156.47±19.67^b^ |
| **K** | 21249.27±1498.62^a^ | 21202.72±717.34^a^ | 18934.77±577.01^b^ |
| **Ca** | 37579.37±8846.44^a^ | 32259.52±5600.36^a^ | 35605.04±10322.33^a^ |
| **Sc** | 15.27±1.02^b^ | 17.25±0.87^a^ | 15.36±1.01^b^ |
| **Ti** | 5886.79±274.29^a^ | 5843.51±206.17^a^ | 5461.00±165.34^b^ |
| **V** | 138.84±3.43^b^ | 146.46±2.98^a^ | 146.29±7.24^a^ |
| **Cr** | 140.87±4.33^b^ | 149.3±2.41^a^ | 135.56±5.40^c^ |
| **Mn** | 722.19±28.06^a^ | 680.88±41.17^a^ | 527.42±70.52^b^ |
| **Fe** | 51737.78±1757.49^b^ | 54308.33±1160.7^a^ | 50235.29±2159.24^b^ |
| **Co** | 21.37±1.42^b^ | 23.00±1.22^a^ | 20.57±1.76^b^ |
| **Ni** | 98.76±3.01^b^ | 109.40±3.33^a^ | 99.38±3.59^b^ |
| **Cu** | 33.88±1.79^b^ | 37.88±1.51^a^ | 34.70±1.40^b^ |
| **Zn** | 113.13±2.72^b^ | 117.81±2.60^a^ | 109.09±3.77^c^ |
| **Se** | 4.89±1.92^b^ | 7.49±0.54^a^ | 5.38±1.08^b^ |
| **Zr** | 160.79±9.4^a^ | 159.97±8.09^a^ | 147.72±4.24^b^ |
| **Ba** | 298.85±12.1^a^ | 301.89±11.52^a^ | 272.10±8.70^b^ |
| **Pb** | 16.44±1.19^a^ | 15.88±1.33^a^ | 13.62±1.33^b^ |
| **PLI†** | 1.19±0.03^b^ | 1.24±0.02^a^ | 1.15±0.03^c^ |
| **Dc††** | 12.3±0.36^b^ | 12.85±0.22^a^ | 11.90±0.30^c^ |
| † PLI = (CF1 × CF2 × CF3 × . . . × CFn)^1/n^; CF: contamination factor | | | |
| ††Dc = $\sum_{i=1}^{n} \mathrm{CF}_{i}$ | | | |

**
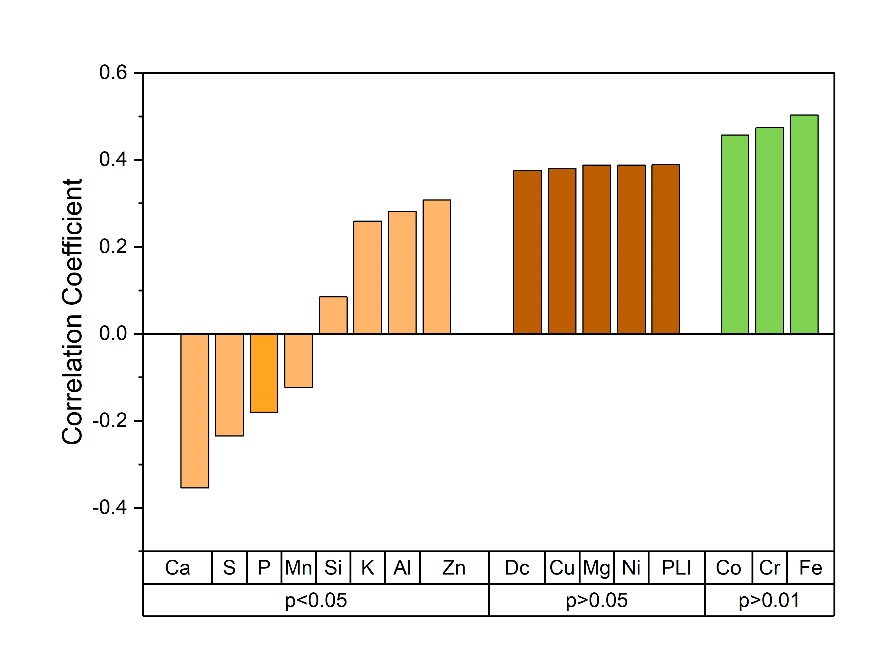
**

**Fig. S1 Pearson correlation coefficient between MAR and selected elemental concentrations, as well as PLI and Dc indices, up to 45 cm (n=36). The parameters were grouped by p-level, where p < 0.05, p > 0.05, and p > 0.01 indicate that the correlation was insignificant, significant at levels of 0.05 and 0.01, respectively.**


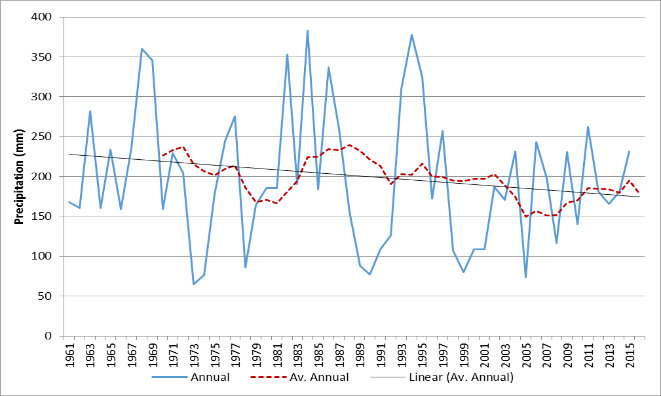

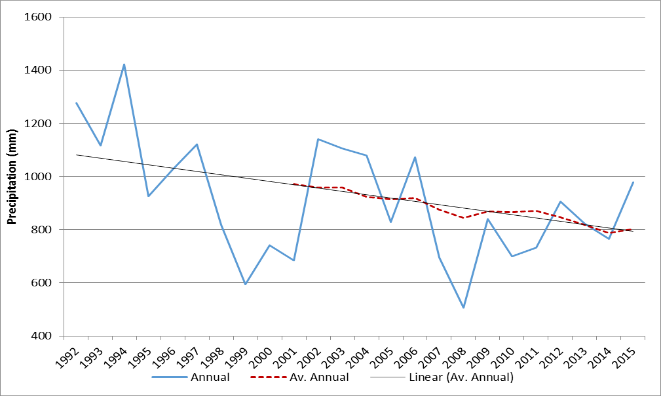


a

b

| **Parameter** | **Precipitation** | | **Maximum temperature** | | **Minimum temperature** | | **Wind** | |
| --- | --- | --- | --- | --- | --- | --- | --- | --- |
| **Station** | **Sanandaj** | **Marivan** | **Sanandaj** | **Marivan** | **Sanandaj** | **Marivan** | **Sanandaj** | **Marivan** |
| **Annual** | decreasing | decreasing | increasing | no trend | increasing | no trend | no trend | no trend |

**Fig. S2** Mean annual precipitation (mm) in Sanandaj (a) and Marivan (b) meteorological stations. 10-year averages were shown in the red dashed line. The trend analysis of various climatic parameters, utilizing the nonparametric Mann-Kendall test and the nonparametric Sen’s method, was also presented for both meteorological stations.


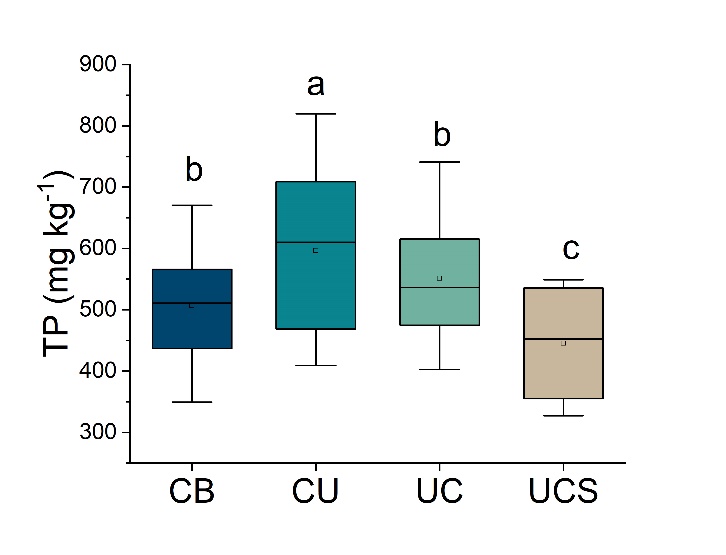

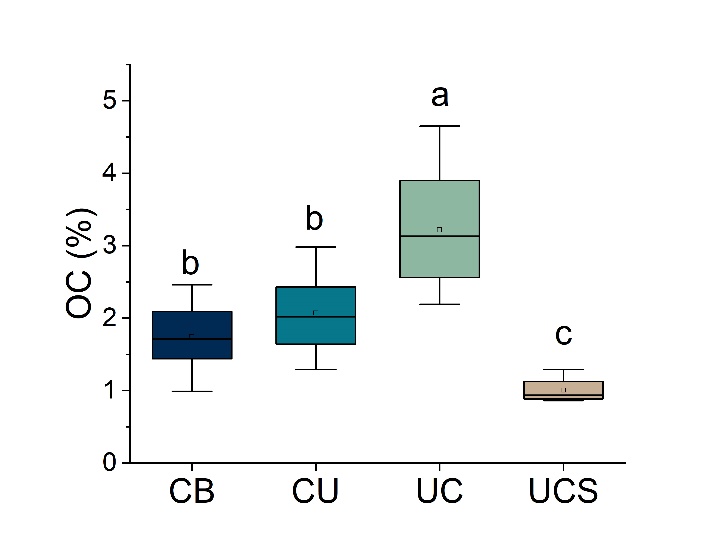


a

b

**Fig. S3** The range of OC (a) and total phosphorus (TP) (b) in soils of the sub-watershed; CB (channel bank), CU (cultivated), UC (uncultivated), and USC (uncultivated subsoil). Different letters indicate significant differences at the p-level < 0.05.

**
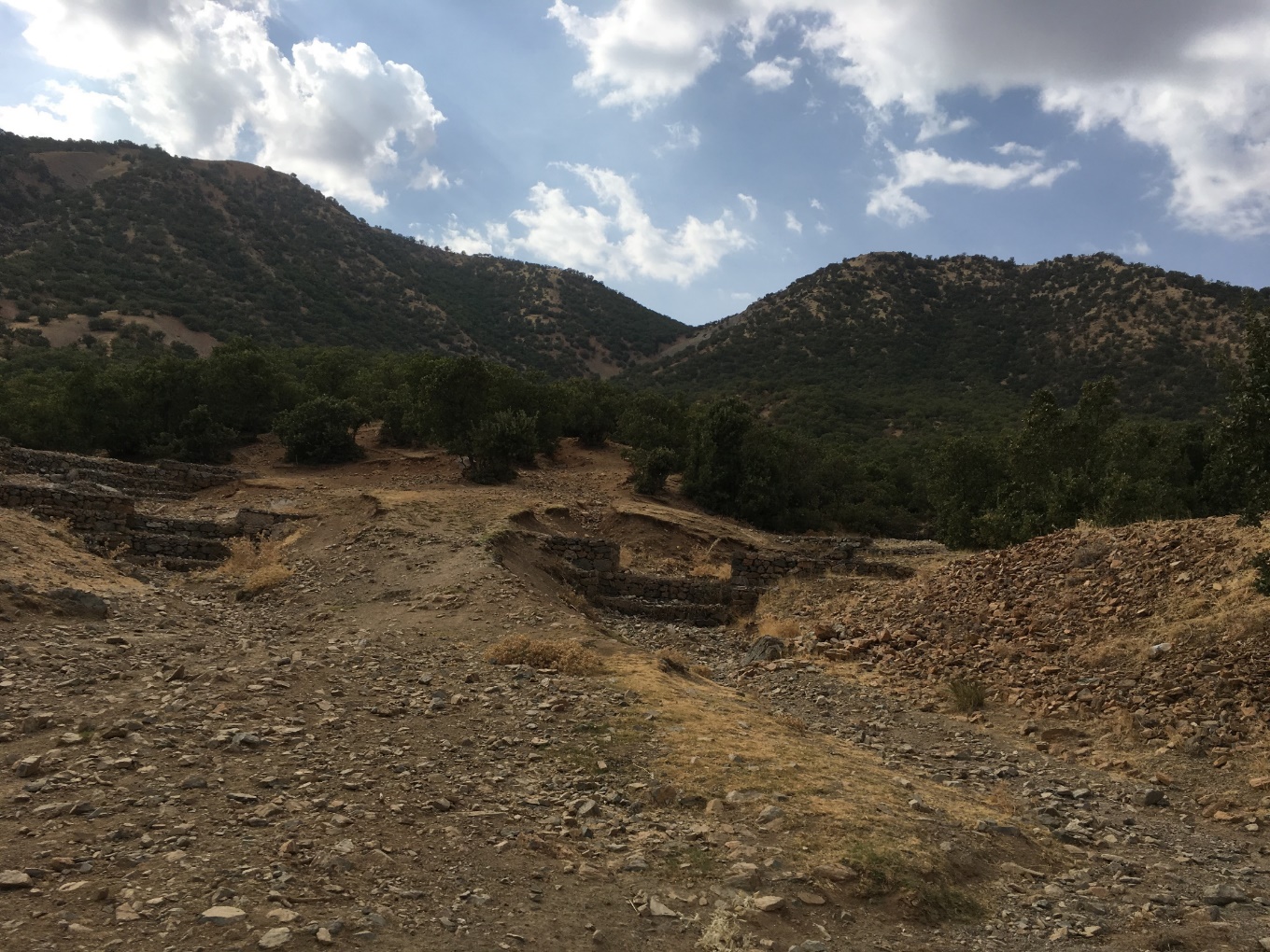

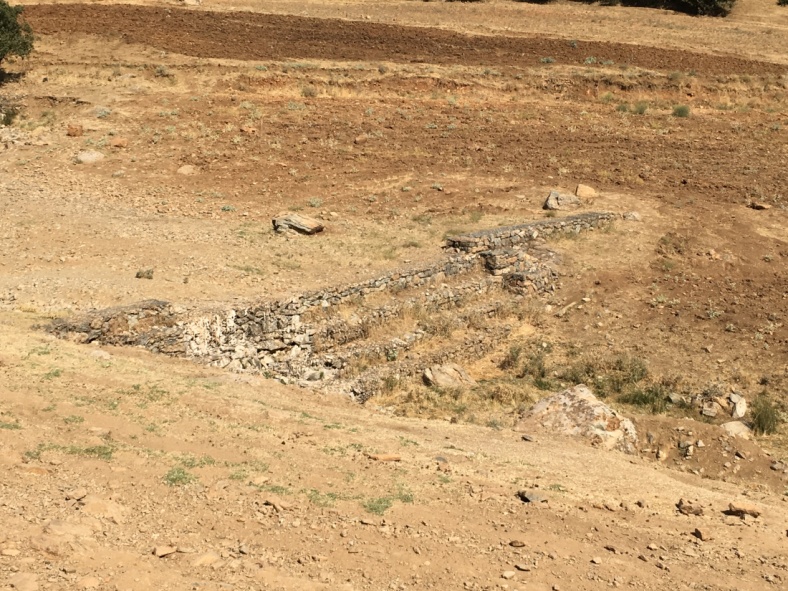
**

**a**

**
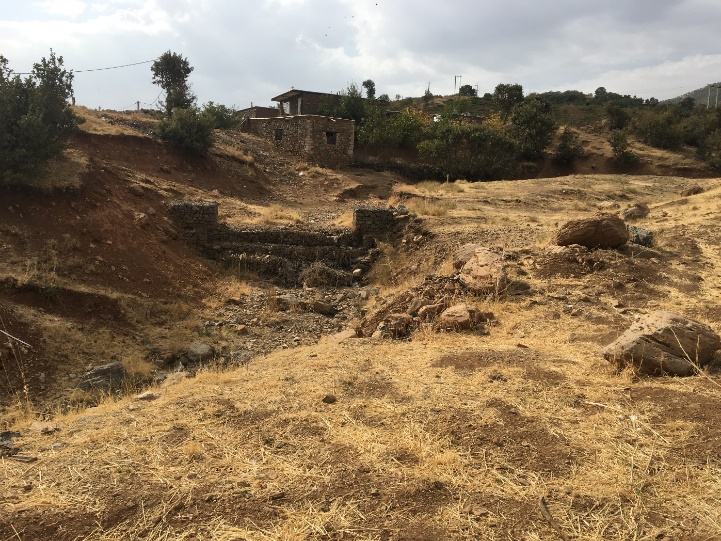
**

**
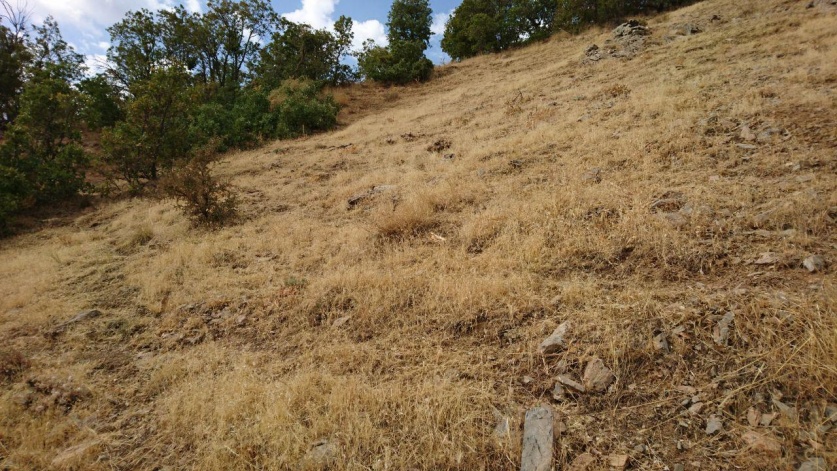

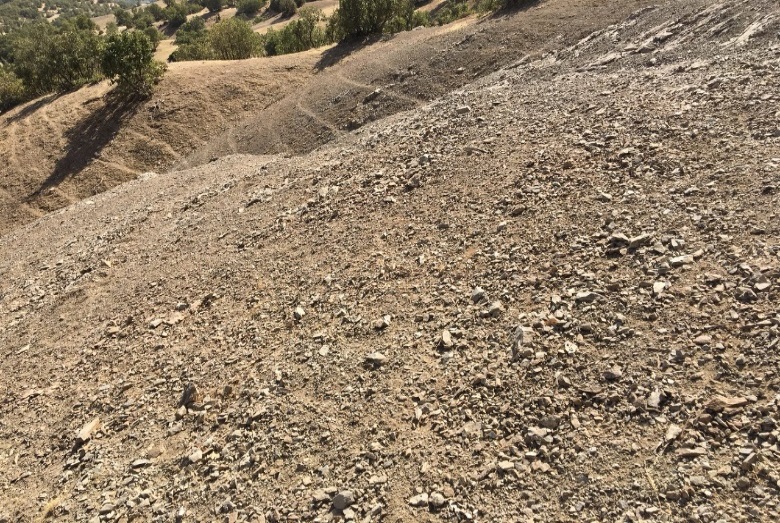
**

**b**

**Fig. S4 Soil conservation practices in the sub-watershed, including numerous check dams (a) and terraces (on steep slopes in forested areas; b) established in 2008-2009 (photos were taken in 2016). Note: most check dams are almost full of sediment.**
